# Hematological Indices in Portal Hypertension: Cirrhosis versus Noncirrhotic Portal Hypertension

**DOI:** 10.3390/jcm7080196

**Published:** 2018-08-02

**Authors:** Abdurrahman Sahin, Hakan Artas, Nurettin Tunc, Mehmet Yalniz, Ibrahim Halil Bahcecioglu

**Affiliations:** 1 Division of Gastroenterology, Department of Internal Medicine, Firat University School of Medicine, 23200 Elazig, Turkey; nurettin@firat.edu.tr (N.T.); mehmetyalniz@hotmail.com (M.Y.); ihbahcecioglu@yahoo.com (I.H.B.); 2 Department of Radiology, Firat University School of Medicine, 23200 Elazig, Turkey; hakanartas@yahoo.com

**Keywords:** hematological indices, portal hypertension, cirrhosis, noncirrhotic portal hypertension, neutrophil to lymphocyte ratio

## Abstract

Portal hypertension (PHT) leads to several alterations on hematological indices (HI). The aim of the study is to investigate the differences in HI between cirrhotic subjects and subjects who have noncirrhotic PHT (NCPHT). This retrospective study included 328 patients with PHT (239 cirrhosis and 89 NCPHT). Demographic and clinical features, endoscopic and radiological findings, and HI including neutrophil to lymphocyte ratio (NLR) at the time of PHT diagnosis were recorded. Severity of cirrhosis was assessed according to the Child–Turcotte–Pugh (CTP) classification and Model for End-Stage Liver Disease (MELD) scores. Hematological abnormalities were found in 92.5% of cirrhotic patients and in 55.1% of patients with NCPHT (*p* < 0.001). While thrombocytopenia was the most common HI in patients with cirrhosis, anemia was the most prevalent HI in NCPHT group. In the cirrhotic group, the NLR was the only parameter to differentiate each CTP group from two others. The NLR value increased with the severity of cirrhosis (2.28 ± 0.14 in CTP-A, 2.85 ± 0.19 in CTP-B and 3.26 ± 0.37 in CTP-C). The AUROC of NLR was 0.692 for differentiating compensated cirrhotic patients from decompensated. Hematological abnormalities are more prevalent and more severe in cirrhotic patients compared to patients with NCPHT. NLR may be used to assess the severity of cirrhosis.

## 1. Introduction

Portal hypertension (PHT) is intravascular pressure in the portal system and defined as a hepatic venous pressure gradient (HVPG) ≥5 mmHg. It is clinically significant at 10 mmHg and predicts development of varices at 10 to 12 mmHg [1,2,3]. The main cause of PHT is cirrhosis. Several disorders other than cirrhosis cause PHT and they are collectively called noncirrhotic PHT (NCPHT) [4]. PHT is responsible for clinical decompensation and most severe complications in cirrhosis and leading cause of death and liver transplantation [2,5].

PHT leads to developments of portosystemic collateral and varices, ascites, spontaneous bacterial peritonitis, and hepatic encephalopathy in cirrhosis [6]. PHT related to cirrhosis and NCPHT exhibits similar clinical features, like splenomegaly, ascites, variceal bleeding, and encephalopathy. It is problematic to differentiate cirrhotic PHT from NCPHT in the clinical ground irrespective from etiological factor.

The measurement of the HVPG is the gold standard technique for the detection of PHT. However, its invasiveness and limited availability preclude the widespread use of this technique for the diagnosis and monitoring of PHT [7]. Instead, several noninvasive tools have been used, including laboratory tests and imaging techniques for the evaluation of PHT. The imaging modalities in the evaluation of PHT consist of ultrasound (US), computed tomography (CT), and magnetic resonance (MR)-based methods, endoscopic US, and angiography. Doppler US is the most widely used imaging modality. On the other hand, no reliable laboratory test has been detected in the assessment of PHT until today.

In recent years, a simple, inexpensive, and widely available test, the full blood count (hemogram), has drawn the attention of several investigators. Indices derived from leukocyte subgroups and platelets have been linked to indicators of systemic inflammation and carcinogenesis. One of the most studied indices is the neutrophil to lymphocyte ratio (NLR). On the other hand, hypersplenism related with PHT leads to cytopenia in one to three major formed elements of blood [8]. There is scarce data about the effect of hypersplenism on hematological indices like NLR and PLR. We aimed in this study to investigate the alterations of hematological parameters in PHT-related hypersplenism, and also the clinical value of NLR in PHT.

## 2. Experimental Section

### 2.1. Study Population

This retrospective observational cohort study was performed at the Elazig Firat University Hospital. After the approval of the study protocol by the Institutional Review Board of Firat University Medicine Faculty, portal vein Doppler US reports between January 2010 and January 2017 were reviewed and the cases of PHT related with cirrhosis or NCPHT were recruited to the study. The subjects with normal US findings and those who had splenomegaly not related with PHT, like acute infection, benign or malign hematological disorders, collagen vascular disease, previous or concomitant other malignancies, and past infection that results in splenomegaly were excluded from our study. From 843 patients, 405 patients were diagnosed with PHT. After the exclusion of subjects who have incomplete clinical, endoscopic, radiological and laboratory data, 328 patients were included in the final analysis. 

### 2.2. Clinical, Laboratory and Radiological Assessments

Demographic information, clinical data, and laboratory data were collected from hospital records. The demographics collected included age, sex, the presence of ascites, endoscopic findings, past variceal bleeding history of the patients with cirrhosis and NCPHT. In patients with cirrhosis, Child–Turcotte–Pugh (CTP) classification at the time of the performance of the Portal Doppler US radiological assessment was obtained from hospital records and patients with cirrhosis belonging in CTP-A class were accepted compensated, whereas those in CTP-B and CTP-C class were assessed decompensated. Additionally, the Model for End-Stage Liver Disease (MELD) scores of patients with cirrhosis at the time of radiological assessment was also noted.

At endoscopic examination, esophageal varices were classified as varices appearing as slight protrusion above mucosa, which can be depressed with insufflations (Grade 1), varices occupying <50% of the lumen (Grade 2), and varices occupying >50% of the lumen and which are very close to each other with confluent appearance (Grade 3). Classification of gastric varices was as follows: continuation of esophageal varices into the lesser curvature (gastroesophageal varices Type 1), esophageal and fundal varices in continuity with the greater curvature (gastroesophageal varices Type 2), fundal varices in the cardia in the absence of esophageal varices (isolated gastric varices Type 1), and fundal varices in the stomach outside of the cardiofundal region or first part of duodenum (isolated gastric varices Type 2) [9].

The laboratory analyses at the time of the radiological assessment included a complete blood count, including hemoglobin, white blood cell, neutrophil counts, lymphocytes, and platelets. Cytopenia was defined as the presence of anemia and/or leukopenia and/or thrombocytopenia. Anemia was defined as hemoglobin values under 13.5 g/dL for men and 12 g/dL for women. Leukopenia was accepted under 4000/mm^3^ of white blood cell count, and thrombocytopenia was the value of platelet count under 150,000/mm^3^. These values were determined according to the definitions used by previous studies and guidelines [10,11,12]. The NLR was obtained by neutrophil count divided by lymphocyte count [13]. Total bilirubin, INR, albumin, and creatinine values of the patients were also recorded.

### 2.3. Statistical Analysis

Statistical analyses were carried out using the Statistical Package for the Social Sciences (SPSS) (version 22; SPSS Inc., Chicago, IL, USA). The variables were analyzed using histogram, the Kolmogorov–Smirnov test, and Shapiro–Wilk’s test to check the distribution of variables. Categorical variables were displayed as numbers and percentages. Continuous variables with a normal distribution were represented as mean ± standard deviation, those with a non-normal distribution and ordinal variables were described as median ± standard error or interquartile range. A chi-square test was used for categorical variables. The Student *t*-test was used for normally distributed parameters, whereas for non-normally distributed factors, the Mann–Whitney U test was used for comparison between two groups. For the assessment of correlation between NLR and cirrhosis severity indices, Spearman’s correlation analysis was conducted. The ability of NLR in cirrhotic group to differentiate compensated cases (CTP-A) from decompensated cases (CTP-B and C) was also investigated by receiver operating characteristics (ROCs) analysis. All statistical analyses were performed using the SPSS, version 22 (IBM Corporation, Armonk, NY, USA). A *p* value of <0.05 was considered statistically significant.

## 3. Results

### 3.1. Characteristics of Patients

A total of 239 patients with cirrhosis (134 male, 105 female) and 89 patients who had NCPHT (51 male, 38 female) were recruited to the study. The mean age of patients with cirrhosis was higher than of patients with NCPHT (58.3 ± 13.9 vs. 46.5 ± 17.3, *p* < 0.001). In the cirrhotic group, 91 patients were classified as CTP-A, 88 patients as CTP-B, and 60 patients as CTP-C. The median MELD score of patients with cirrhosis was 8 (IQR, 4–12). Demographic, clinical, and radiological features were given in Table 1. The most common etiological factors for cirrhosis were chronic hepatitis B (CHB) in 57 patients (23.8%), chronic hepatitis D in 20 patients (8.3%), chronic hepatitis C in 16 patients (6.7%), nonalcoholic steatohepatitis in 31 patients (13%), alcoholic cirrhosis in 15 patients (6.3%), autoimmune hepatitis in 11 patients (4.6%), and primary biliary cholangitis in 9 patients (3.8%). On the other hand, any etiological factor was not defined in 70 subjects (29.3%) and they were classified as cryptogenic cirrhosis.

### 3.2. PHT and Related Complications

Splenomegaly was detected in 200 patients with cirrhosis (87.0%) and 61 patients with NCPHT (70.1%) (*p* < 0.001). Median spleen length was also higher in the cirrhotic group than the NCPHT group (150 ± 1.8 mm vs. 140 ± 3.5 mm, *p* = 0.002). On the other hand, portal vein thrombosis was more prevalent in the NCPHT group (*n* = 33) than in patients with cirrhosis (*n* = 47) (37.1% vs. 19.7%, *p* = 0.004).

While varices in esophagus and/or stomach were evident in 200 patients with cirrhosis (83.7%), only 45 patients with NCPHT (50.6%) had esophageal and/or gastric varices (*p* < 0.001). The percentage of patients who had esophageal varices was higher in patients with cirrhosis (198 patients, 82.8%) than in patients with NCPHT (39 patients, 43.9%) (*p* < 0.001). On the other hand, there was no statistically significant difference in terms of the presence of gastric varices between cirrhotic patients (*n* = 33) and patients with NCPHT (*n* = 14) (13.8% vs. 15.7%, *p* = 0.737). Past variceal bleeding was more prevalent among patients with cirrhosis (56, 28.0%) compared to patients with NCPHT (3, 6.6%) (*p* < 0.001).

Similarly, the presence of ascites was higher in cirrhotic group (*n* = 142) than patients with NCPHT (*n* = 12) (59.4% vs. 13.8%, *p* < 0.001). While 50 patients (20.9%) in the cirrhotic group had past hepatic encephalopathy, none of the patients with NCPHT had hepatic encephalopathy (*p* < 0.001). The comparison of both groups in terms of demographic, clinical, and PHT-related complications were given in Table 1.

### 3.3. Comparison of Hematological Indices between Cirrhosis and NCPHT

**Figure 1:**
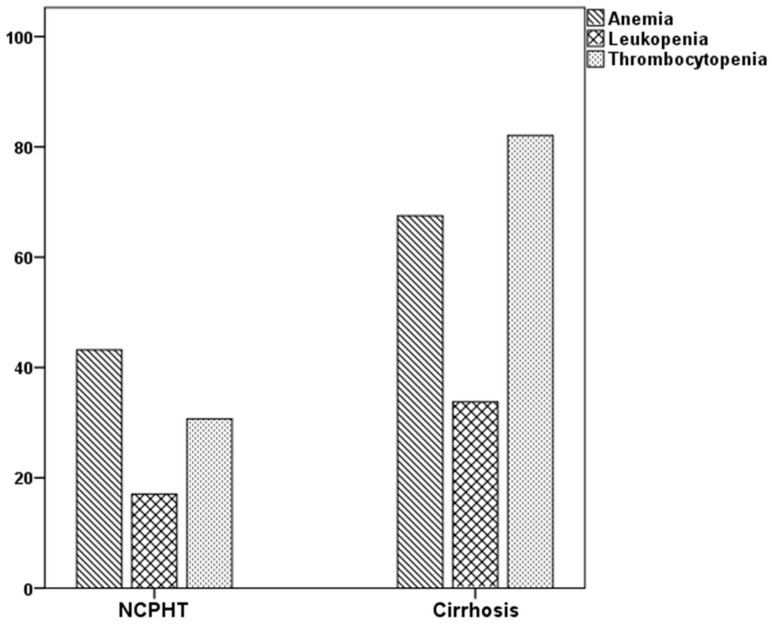
Percentage of cytopenias in patients with portal hypertension.

The rates of abnormalities in the three major blood forms, in at least one were higher in patients with cirrhosis than the NCPHT group. While 44.9% of patients with NCPHT had normal hematological indices, only 7.5% of patients in the cirrhotic group had above the cutoff values in all three lineages (*p* < 0.001). The rates of hematological abnormalities were given in Table 2 and Figure 1. Thrombocytopenia, which was the most common abnormality among patients with cirrhosis (82.7%), was more prevalent compared to the patients with NCPHT (30.3%) (*p* < 0.001). Anemia, which was the most common hematological abnormality in the NCPHT group with 37.1%, was also higher among patients with cirrhosis (61.5%) (*p* < 0.001). Leukopenia was the least common abnormality in patients with cirrhosis (33.9%) and NCPHT (16.9%) (*p* = 0.03).

Median leukocyte, neutrophil, and lymphocyte counts were higher in patients with NCPHT compared to patients with cirrhosis (5840 ± 205 vs. 4585 ± 126, *p* < 0.001; 3420 ± 156 vs. 2845 ± 88, *p* = 0.005; 1390 ± 76 vs. 1075 ± 37, *p* < 0.001, respectively). The median thrombocyte count was also higher in the NCPHT group (202,000 ± 10,820 vs. 103,000 ± 3600, *p* < 0.001). Mean hemoglobin value was higher in patients with NCPHT than patients with cirrhosis (13.0 ± 0.2 vs. 11.7 ± 0.2, *p* < 0.001). Median NLR did not differ between groups (2.71 ± 0.14 for cirrhosis, 2.47 ± 0.19 for NCPHT, *p* = 0.125).

In the subgroup analysis of study participants by age 50, median leukocyte, neutrophil, and lymphocyte counts, median thrombocyte counts, and mean hemoglobin values were also higher; mean spleen sizes were lower in the NCPHT group than the cirrhotic group for both ages <50 and ≥50 years (for all, *p* < 0.05) (Table 3). On the other hand, median NLR values were not different in both subgroups.

### 3.4. Influences of Gender and Age on Hematological Indices

A total of 185 male and 143 female subjects were recruited in the final analysis. The mean age, spleen size, thrombocyte count, NLR, albumin, international normalized ratio (INR), and bilirubin were not different between groups. Leukocyte, neutrophil and lymphocyte counts, and hemoglobin values were lower in female subjects than male subjects (for all, *p* < 0.001). In the NCPHT group, age, spleen size, neutrophil count, thrombocyte count, NLR, albumin, and INR did not differ between females and males (for all, *p* > 0.05). On the other hand, leukocyte and lymphocyte counts, hemoglobin values, and bilirubin were lower in females compared to males (*p* = 0.003, *p* = 0.006, *p* < 0.001 and *p* = 0.007, respectively). In the cirrhotic group, the mean age was lower in males (56.7 ± 13.3 vs. 60.3 ± 14.2, *p* = 0.042). Leukocyte, neutrophil and lymphocyte counts, and thrombocyte counts and hemoglobin values were also lower in females (*p* < 0.001, *p* < 0.001, *p* = 0.001, *p* < 0.001, and *p* = 0.016, respectively). Spleen size, NLR, albumin, INR, and bilirubin were not different between groups (for all, *p* > 0.05).

In the analysis of the entire group, age was negatively correlated with spleen size (*r^2^* = −0.164, *p* = 0.003), lymphocyte count (*r^2^* = –0.118, *p* = 0.033), hemoglobin (*r^2^* = −0.221, *p* < 0.001), and albumin (*r^2^* = −0.401, *p* < 0.001). On the other hand, age was positively correlated with NLR (*r^2^* = 0.140, *p* = 0.011). In the NCPHT group, age was only correlated with albumin (*r^2^* = −0.264, *p* = 0.012). Among patients with cirrhosis, age was negatively correlated with spleen size (*r^2^* = −0.250, *p* < 0.001), hemoglobin (*r^2^* = –0.166, *p* = 0.010), and albumin (*r^2^* = –0.299, *p* < 0.001). Thrombocyte count (*r^2^* = 0.206, *p* = 0.001) and NLR (*r^2^* = 0.152, *p* = 0.019) were found as positively correlated parameters with age in the cirrhotic group.

### 3.5. Splenomegaly and Hematological Indices

Splenomegaly was detected in 269 subjects. Leukocyte, neutrophil and lymphocyte counts, thrombocyte counts, and mean hemoglobin values were lower in patients who had splenomegaly (for all, *p* < 0.05) (data not shown). On the other hand, median NLR value was higher in patients with splenomegaly than in patients without splenomegaly (2.69 ± 0.13 vs. 2.28 ± 0.24, *p* = 0.032).

### 3.6. Comparison of Hematological Indices in Cirrhosis

**Figure 2:**
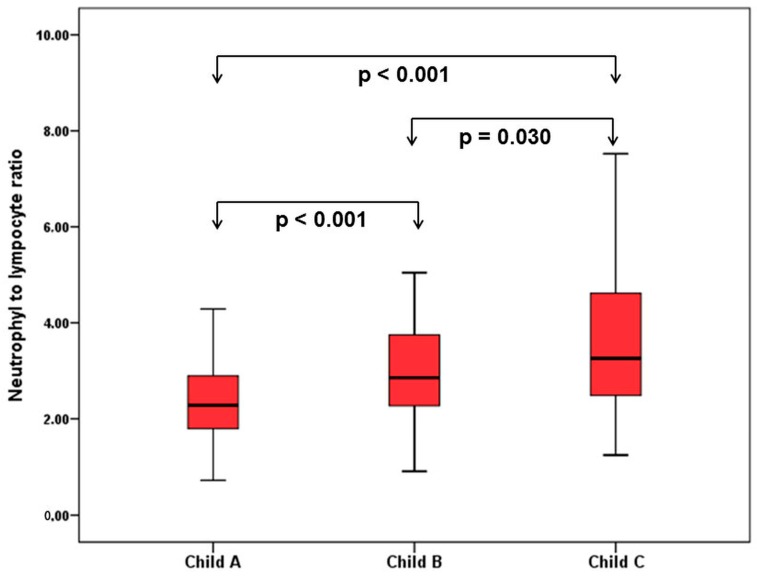
Comparison of NLR in patients with cirrhosis according to Child–Turcotte–Pugh (CTP) classification.

**Figure 3:**
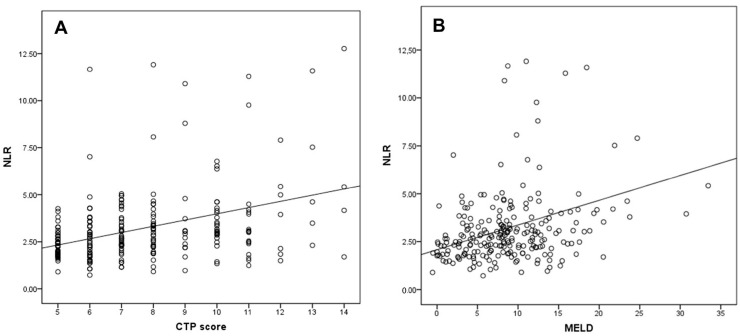
Correlation of the NLR with (**A**) CTP score and (**B**) MELD score.

**Figure 4:**
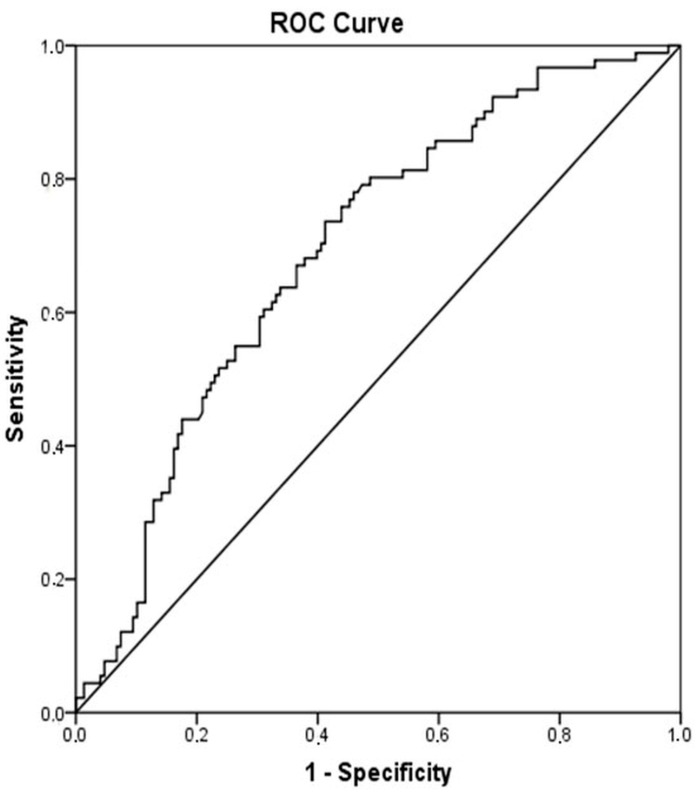
Receiver operator curves of NLR for differentiating decompensated cirrhotic subjects (CTP-B and CTP-C) from compensated cirrhotic subjects (CTP-A). Required changes have been made on Figure 2.

When patients with cirrhosis were divided into three groups according to CTP classification, the comparisons of hematological indices were given in Table 4. The NLR was lower in CTP-A (2.28 ± 0.14) than both CTP-B (2.85 ± 0.19, *p* < 0.001) and CTP-C (3.26 ± 0.37, *p* < 0.001). Moreover, NLR was also lower in CTP-B compared to CTP-C (*p* = 0.030) (Figure 2). A significant positive correlation was found between NLR and CTP score (*r^2^* = 0.345, *p* < 0.001) and MELD score (*r*^2^ = 0.294, *p* < 0.001) (Figure 3). The AUROC of NLR was 0.692 (95% CI, 0.624–0.759; *p* < 0.001) differentiating compensated cirrhotic patients from decompensated patients at a cutoff value of 2.33 with 55% sensitivity and 74% specificity (Figure 4). While several differences were detected in hematological indices of cirrhotic patients according to CTP groups, there was no difference in terms of spleen length (for all, *p* > 0.05).

## 4. Discussion

In the present study, the effect of PHT on hematological indices was examined. To our knowledge, this retrospective study is the first study comparing hematological indices in cirrhosis and NCPHT. Until now, peripheral cytopenia has been investigated in groups of both cirrhosis and NCPHT, separately [12,14,15,16]. Differences of cutoff values in three lineages, selection of specific patient populations, such as studies on patients with compensated cirrhosis or those undergoing splenectomy for NCPHT, and the methodology that was used, like prospective or retrospective, result in the variability in a wide range of hematological abnormalities in previous studies.

Hypersplenism is defined as the presence of splenomegaly and peripheral cytopenia in the event of normal or hypercellular bone marrow [14]. Splenomegaly is the principal factor in PHT for the development of cytopenia. Lv et al. demonstrated that the main cause of peripheral cytopenia in cirrhotic patients undergoing splenectomy was splenomegaly with an 80.5 percentage [17]. In earlier studies, hypersplenism was detected in a wide range from 15 to 70% of patients with cirrhosis, splenomegaly from 36 to 92%, leukopenia from 11 to 35%, and thrombocytopenia from 11 to 20% [14]. In a prospective study on compensated cirrhotic subjects, thrombocytopenia was evident in 77.9% of participants, leukopenia in 23.5%, and anemia in 21.1% at baseline [12]. Bashour et al. demonstrated in patients with nonalcoholic cirrhosis that thrombocytopenia was the most common hematological abnormality with a 64% [15]. An interesting finding of the mentioned study was the lower rate of leukopenia (5%) that was defined as <3500 of WBC count. Another study, which was conducted on 330 patients undergoing splenectomy for PHT and splenomegaly related with nonalcoholic cirrhosis, demonstrated that monocytopenia was evident in 30%, bicytopenia in 36%, and pancytopenia in 34% of patients [18]. In another series that consisted of 64 patients with NCPHT undergoing surgery for hypersplenism, the rates of thrombocytopenia, leukopenia, and anemia at baseline were 81%, 57%, and 59%, and values in all of the three lineages reached to above the cutoff values within the first month of surgery [16].

An interesting finding of the present study was that the percentage of hematological abnormalities was higher in patients with cirrhosis in all of three lineages. Beside hematological findings, higher prevalence of PH-related clinical findings, including varices, ascites, and hepatic encephalopathy suggests that the effect of PHT was more pronounced in cirrhotic patients than in the noncirrhotic group. Thrombocytopenia has been shown to be the most common hematological abnormality in cirrhotic patients and related with the severity of the liver disease [12,15]. It is established that splenic sequestration is the leading cause of thrombocytopenia in cirrhosis [19]. On the other hand, PHT has a limited role in the development of leukopenia and anemia. Alterations in bone marrow-stimulating factors, bone marrow suppression by toxins, and ongoing blood loss are other factors that contribute to the development of cytopenia in cirrhosis [19]. In NCPHT, anemia is the most common hematological abnormality, followed by thrombocytopenia and leucopenia [8]. Anemia is related to variceal bleeding, iron deficiency, and hypersplenism. In concordance with the literature, we showed in the current study that, while anemia was the most common abnormality among patients with NCPHT, thrombocytopenia was the major abnormality among cirrhotic subjects.

The second important observation of the current study was that the NLR was the only parameter to differentiate each CTP group from the two others. The NLR value gradually increased from CTP-A to CTP-C and, thus, was correlated with the severity of cirrhosis. There are some studies investigating NLR value among cirrhotic subjects [20,21,22]. Biyik et al. showed that cirrhotic patients who had a NLR value over 2.72 had significantly lower survival [22]. In another study, NLR was found an independent predictor of one-month mortality among CTP-C class cirrhotic subjects [20]. Zhang et al. also demonstrated on cirrhotic patients related with CHB that CTP-C class patients have higher leukocyte and neutrophil counts and lower lymphocyte counts than patients in CTP-B class and CTP-A class [23]. In the mentioned study, NLR was found an independent predictor of three-month mortality and was associated with the severity of cirrhosis [23]. In accordance with previous studies, the present study showed that NLR value is correlated with the CTP and MELD scores. Moreover, we showed that NLR is a unique hematological parameter to discriminate cirrhotic patients according to CTP class.

This study has several limitations. First, this is a retrospective observational study, and the course of diseases and outcome cannot be established in the present study. Further long-term follow-up studies are warranted to explore the predictive value of hematological parameters on the course of diseases and outcome. We used only one hemogram parameter in the present study. However, several factors influence these parameters, especially leucocyte and its subgroups. Thus, hematological measurements, especially NLR values, cannot be controlled with the cross-sectional method. Thirdly, the gold standard method to establish PHT is HPVG measurement. On the other hand, due to the retrospective nature of the current study, PHT was defined according to portal Doppler US measurements. Interobserver variability may influence the results of the present study.

## 5. Conclusions

In conclusion, hematological abnormalities are more common in cirrhosis compared to NCPHT. Correlation of NLR with CTP and MELD indicates that an elevated NLR establishes more severe disease in cirrhosis. Therefore, serial measurements of NLR may be helpful to monitor the course of disease in patients with cirrhosis.

## Figures and Tables

**Table 1:** Demographic and clinical features of patients with cirrhosis and patients with noncirrhotic portal hypertension (NCPHT).

	Cirrhosis (*n* = 239)	NCPHT (*n* = 89)	*p*
Age, years	58.3 ± 13.9	46.5 ± 17.3	<0.001
Sex, male/female	134/105	51/38	0.841
Varices, *n*(%)	200 (83.7%)	45 (50.6%)	<0.001
Esophageal varices, *n*(%)	198 (82.8%)	39 (43.9%)	<0.001
Grade 1, *n*(%)	45 (18.8%)	15 (16.9%)	
Grade 2, *n*(%)	86 (36.0%)	15 (16.9%)	
Grade 3, *n*(%)	64 (26.8%)	9 (10.1%)	
Gastric varices, *n*(%)	33 (13.8%)	14 (15.7%)	0.737
GOV-1, *n*(%)	10 (4.2%)	4 (4.5%)	
GOV-2, *n*(%)	8 (3.3%)	2 (2.2%)	
IGV-1, *n*(%)	15 (6.3%)	8 (9.0%)	
IGV-2, *n*(%)	0	0	
Variceal bleeding, *n*(%)	56 (28.0%)	3 (6.6%)	<0.001
Ascites, *n*(%)	142 (59.4%)	12 (13.8%)	<0.001
Grade 1, *n*(%)	44 (18.4%)	5 (5.6%)	
Grade 2, *n*(%)	46 (19.2%)	6 (6.7%)	
Grade 3, *n*(%)	52 (21.8%)	0	
Hepatic encephalopathy, *n*(%)	50 (20.9%)	0	<0.001
Splenomegaly, *n*(%)	208 (87.0%)	61 (70.1%)	<0.001
Spleen, mm (median ± SE)	150 ± 1.8	140 ± 3.5	0.002
Portal vein thrombosis, *n*(%)	47 (19.7%)	33 (37.1%)	0.004
Child–Turcotte–Pugh class			
Class A, *n*(%)	91 (38.1%)		
Class B, *n*(%)	88 (36.8%)		
Class C, *n*(%)	60 (25.1%)		
Model for End-Stage Liver Disease (MELD), median (IQR)	8 (4–12)		

GOV, gastroesophageal varices; IGV, isolated gastric varices.

**Table 2:** Hematological indices in patients with cirrhosis and patients with NCPHT.

	Cirrhosis (*n* = 239)	NCPHT (*n* = 89)	*p*
Cytopenia, *n*(%)	221 (92.5%)	49 (55.1%)	<0.001
Normal, *n*(%)	18 (7.5%)	40 (44.9%)	
Monocytopenia, *n*(%)	72 (30.2%)	30 (33.7%)	
Bicytopenia, *n*(%)	95 (39.7%)	12 (13.5%)	
Pancytopenia, *n*(%)	54 (22.6%)	7 (7.9%)	
Anemia, *n*(%)	147 (61.5%)	33 (37.1%)	<0.001
Leukopenia, *n*(%)	81 (33.9%)	15 (16.9%)	0.030
Thrombocytopenia, *n*(%)	197 (82.4%)	27 (30.3%)	<0.001
Leukocyte, (×10^3^/uL)	4585 ± 126	5840 ± 205	<0.001
Neutrophil, (×10^3^/uL)	2845 ± 88	3420 ± 156	0.005
Lymphocyte, (×10^3^/uL)	1075 ± 37	1390 ± 76	<0.001
Hemoglobin, g/dL	11.7 ± 0.2	13.0 ± 0.2	<0.001
Platelet, (×10^3^/uL)	103,000 ± 3600	202,000 ± 10820	<0.001
NLR	2.71 ± 0.14	2.47 ± 0.19	0.125
INR	1.33 ± 0.31	1.09 ± 0.14	<0.001
Albumin (g/dL)	3.2 ± 0.7	4.1 ± 0.5	<0.001
Bilirubin,(mg/dL)	1.4 ± 0.23	0.8 ± 0.14	<0.001
Creatinine, (mg/dL)	0.70 ± 0.02	0.78 ± 0.07	0.002

NLR, neutrophil to lymphocyte ratio; INR, international normalized ratio.

**Table 3:** The comparison of hematological indices in subgroups of patients with cirrhosis and patients with NCPHT by age 50.

	<50 Years		*p*	≥50 Years		*p*
	NCPHT (*n* = 51)	Cirrhosis (*n* = 50)		NCPHT (*n* = 38)	Cirrhosis (*n* = 189)	
Age, years	33.7 ± 8.4	37.6 ± 8.5	0.023	63.8 ± 8.9	63.8 ± 8,9	0.995
Leukocyte, (×10^3^/uL)	5750 ± 285	4405 ± 245	0.002	5855 ± 320	4600 ± 145	0.009
Neutrophil, (×10^3^/uL)	3430 ± 210	2730 ± 160	0.006	3320 ± 220	2900 ± 100	0.084
Lymphocyte, (×10^3^/uL)	1360 ± 100	1165 ± 80	0.040	1435 ± 115	1050 ± 40	0.008
Hemoglobin, g/dL	13.1 ± 2.2	12.7 ± 2.5	0.291	12.6 ± 1.9	11.4 ± 2.2	0.002
Platelet, (×10^3^/uL)	204,000 ± 14,300	98,500 ± 6870	<0.001	190,000 ± 16,800	104,000 ± 4160	<0.001
NLR	2.62 ± 0.20	2.35 ± 0.19	0.924	2.40 ± 0.36	2.84 ± 0.16	0.256
Spleen, mm	153 ± 34	167 ± 34	0.041	137 ± 24	151 ± 25	0.001

NLR, neutrophil to lymphocyte ratio.

**Table 4:** Comparison of hematological indices among patients with cirrhosis.

	CTP-A (*n* = 91)	CTP-B (*n* = 88)	CTP-C (*n* = 60)	*p* ^a^	*p* ^b^	*p* ^c^
Leukocyte, (×10^3^/uL)	4400 ± 190	4420 ± 184	5613 ± 293	0.826	0.042	0.020
Neutrophil, (×10^3^/uL)	2780 ± 110	2760 ± 130	3320 ± 220	0.819	0.002	0.005
Lymphocyte, (×10^3^/uL)	1160 ± 70	1050 ± 50	950 ± 60	0.196	0.047	0.336
Hemoglobin, g/dL	12.6 ± 2.2	11.3 ± 2.3	10.7 ± 2.1	0.001	<0.001	0.163
Platelet, (×10^3^/uL)	104,000 ± 5600	108,000 ± 5100	98,000 ± 8870	0.625	0.377	0.202
NLR	2.28 ± 0.14	2.85 ± 0.19	3.26 ± 0.37	<0.001	<0.001	0.030
Spleen, mm	154 ± 31	157 ± 25	153 ± 28	0.512	0.828	0.384

Notes: All values are presented as median ± standard error, unless stated otherwise. *p*^a^ value between CTP-A group and CTP-B group; *p*^b^ value between CTP-A group and CTP-C group; *p*^c^ value between CTP-B group and CTP-C group. NLR, neutrophil to lymphocyte ratio; CTP, Child-Turcotte-Pugh classification.
